# RETRACTION: ENKUR Recruits FBXW7 to Ubiquitinate and Degrade MYH9 and Further Suppress MYH9‐Induced Deubiquitination of β‐Catenin to Block Gastric Cancer Metastasis

**DOI:** 10.1002/mco2.70160

**Published:** 2025-03-11

## Abstract

J. Liu, Z. Liu, W.i Yan, et al., “ENKUR Recruits FBXW7 to Ubiquitinate and Degrade MYH9 and Further Suppress MYH9‐Induced Deubiquitination of β‐catenin to Block Gastric Cancer Metastasis,” *MedComm* 3, no. 4 (2022): e185, https://doi.org/10.1002/mco2.185.

The above article, published online on 25 November 2022 in Wiley Online Library (wileyonlinelibrary.com), has been retracted by agreement between the authors; the journal Editors‐in‐Chief; the Sichuan International Medical Exchange & Promotion Association (SCIMEA); and John Wiley & Sons Australia, Ltd. The retraction has been agreed due to concerns raised by third parties. Further investigation by the publisher has found evidence of image manipulation in Figure 5G. The authors cooperated with the investigation and stated that they were unaware of any manipulation as they did not directly participate in the experiments presented in Figure 5G. They informed the journal that, due to laboratory limitations, an independent company conducted the EMSA experiments and provided them with the digital images of the results instead of original blots. The authors state that they had no reason to doubt the data's authenticity and did not notice irregularities in the images before submission. During the investigation, the authors explained that the company which conducted the EMSA experiments was not the same company stated in the article.

Additionally, a significant portion of the article's conclusions are based on experiments on the cell line BGC‐823, reported as contaminated [1, 2]. The authors provided the STR profile of the cell line used in their study, and this was found to match the problematic cell line BGC‐823 at RRID CVCL_3360. Accordingly, the article must be retracted as the editors and the publisher determined that a significant portion of the article's data is unreliable and consider its conclusions invalid. In light of the concerns identified in the course of the investigation, the authors have agreed to retract the article.

References

1. F. Ye, C. Chen, J. Qin, J. Liu, and C. Zheng, “Genetic Profiling Reveals an Alarming Rate of Cross‐Contamination among Human Cell Lines Used in China,” *The FASEB Journal* 29 (2015): 4268–4272, https://doi.org/10.1096/fj.14‐266718.

2. X. Bian, Z. Yang, H. Feng, H. Sun, and Y. Liu, “A Combination of Species Identification and STR Profiling Identifies Cross‐Contaminated Cells From 482 Human Tumor Cell Lines,” *Scientific Reports* 7 (2017): 9774, https://doi.org/10.1038/s41598‐017‐09660‐w.

J. Liu, Z. Liu, W.i Yan, et al., “ENKUR Recruits FBXW7 to Ubiquitinate and Degrade MYH9 and Further Suppress MYH9‐Induced Deubiquitination of β‐catenin to Block Gastric Cancer Metastasis,” *MedComm* 3, no. 4 (2022): e185, https://doi.org/10.1002/mco2.185.

The above article, published online on 25 November 2022 in Wiley Online Library (wileyonlinelibrary.com), has been retracted by agreement between the authors; the journal Editors‐in‐Chief; the Sichuan International Medical Exchange & Promotion Association (SCIMEA); and John Wiley & Sons Australia, Ltd. The retraction has been agreed due to concerns raised by third parties. Further investigation by the publisher has found evidence of image manipulation in Figure 5G. The authors cooperated with the investigation and stated that they were unaware of any manipulation as they did not directly participate in the experiments presented in Figure 5G. They informed the journal that, due to laboratory limitations, an independent company conducted the EMSA experiments and provided them with the digital images of the results instead of original blots. The authors state that they had no reason to doubt the data's authenticity and did not notice irregularities in the images before submission. During the investigation, the authors explained that the company which conducted the EMSA experiments was not the same company stated in the article.

Additionally, a significant portion of the article's conclusions are based on experiments on the cell line BGC‐823, reported as contaminated [1, 2]. The authors provided the STR profile of the cell line used in their study, and this was found to match the problematic cell line BGC‐823 at RRID CVCL_3360. Accordingly, the article must be retracted as the editors and the publisher determined that a significant portion of the article's data is unreliable and consider its conclusions invalid. In light of the concerns identified in the course of the investigation, the authors have agreed to retract the article.

**References**

1. F. Ye, C. Chen, J. Qin, J. Liu, and C. Zheng, “Genetic Profiling Reveals an Alarming Rate of Cross‐Contamination among Human Cell Lines Used in China,” *The FASEB Journal* 29 (2015): 4268–4272, https://doi.org/10.1096/fj.14‐266718.

2. X. Bian, Z. Yang, H. Feng, H. Sun, and Y. Liu, “A Combination of Species Identification and STR Profiling Identifies Cross‐Contaminated Cells From 482 Human Tumor Cell Lines,” *Scientific Reports* 7 (2017): 9774, https://doi.org/10.1038/s41598‐017‐09660‐w.

